# Harnessing Free Energy From Nature For Efficient Operation of Compressed Air Energy Storage System and Unlocking the Potential of Renewable Power Generation

**DOI:** 10.1038/s41598-018-28025-5

**Published:** 2018-07-02

**Authors:** Gayathri Venkataramani, Velraj Ramalingam, Kishore Viswanathan

**Affiliations:** 1 Institute for Energy Studies, CEG, Anna University, Chennai, 600025 India; 2 Department of Mechanical Engineering, CEG, Anna University, Chennai, 600025 India

## Abstract

Energy storage technologies have gained considerable momentum in the recent years owing to the rising tide of renewables. The deployment of energy storage is a trend set to continue into 2018 and beyond. In the near future, compressed air energy storage (CAES) will serve as an integral component of several energy intensive sectors. However, the major drawback in promoting CAES system in both large and small scale is owing to its minimum turn around efficiency. In the present work the major drawbacks associated with various existing configurations of CAES system are analysed. Interesting results of Isothermal CAES system are obtained through the present analysis to generate additional output energy compared to the supplied input by harnessing the free energy from the natural water bodies/ocean to enhance the overall turnaround efficiency of the system. The optimum operational characteristics of charging and discharging cycles are also addressed. In the present energy scenario, increasing the percentage of renewable energy (RE) share in the power generation is quite challenging since RE based power generation is intermittent in nature. The integration of energy storage technologies with RE source is imperative as it mitigates the intermittency of available energy. However, the development of efficient energy storage systems is one of the prime challenges in the promotion of renewable energy in a large scale. Among the various storage systems, electrochemical battery storage and pumped hydro storage (PHS) have attracted the commercial market. However, the shorter cycle life makes the battery storage more expensive and the PHS systems involves certain geographical and site constraints. Beyond the said storage systems, compressed air energy storage system which is one of the technically proven system has not been targeted the commercial market owing to its lower turnaround efficiency. Hence, the motivation behind the present research is towards developing efficient CAES configuration with higher turnaround efficiency thereby attaining economic feasibility and sustainability.

## Introduction

Kittner *et al*.^1^ deployed the various strategies for the emerging energy storage technologies and made a clear route towards cost effective low carbon electricity. In the recent years, bulk energy storages are gaining large momentum in order to improve the grid stability and to avoid transmission congestion issues. Compressed air energy storage (CAES) is considered as one of the promising large scale energy storage systems with attractive economic benefits. While, discussing the principle of operation, the energy is stored in the form of compressed air by operating a compressor during off peak hours with RE sources and the stored compressed air is released during peak hours through an expander and the electrical energy is generated using an alternator. However, it is well known that in the entire energy transfer and conversion process, the considerable energy loss associated with all the three phases of storage system (charging, discharging and storage) are inevitable.

Hartmann *et al*.^2^ analysed the efficiency of one full charging and discharging cycle of several adiabatic compressed air energy storage configurations. They concluded that the key element for improving the efficiency is to develop a high temperature thermal storage and temperature resistant material for compressors. Kushnir *et al*.^3^ studied the thermodynamic response of underground cavern reservoirs for the analysis of charge/discharge cycles of compressed air energy storage plants. Based on the mass and energy conservation equations, numerical and approximate analytical solutions for the air cavern temperature and pressure variations were derived. Audrius *et al*.^4^ conducted exergy and exergoeconomic analysis of a CAES system with and without Thermal Energy Storage (TES) and found an increase in energy efficiency to 86% and exergy efficiency to 55.8% for the CAES-TES system in comparison with CAES system alone, which reported energy efficiency of 48% and exergy efficiency of 50.1%. Nejad *et al*.^5^ undertook thermodynamic analysis of a wind integrated CAES system and they revealed the fact that the energy conversion process could be adiabatic or isothermal. The major parameters in their analysis were storage pressure, temperature and tank volume (TV). Li *et al*.^6^ proposed a novel micro trigeneration based compressed air system with thermal energy storage technologies. They have also performed the thermodynamic analysis and found that the average comprehensive efficiency is around 50% and 35% in winter and summer respectively that appears to be much higher than the conventional trigeneration system. A near 4 MWh underwater CAES system was numerically simulated to obtain optimal system configuration and the maximum round trip efficiency^7–9^. In such systems, the accumulators are placed underwater utilizing the hydrostatic pressure exerted by the surrounding water bodies^10,11^. Nielsen *et al*.^12^ proposed a concept of isobaric adiabatic CAES system integrated with combined cycle. Houssainy *et al*.^13^ performed thermodynamic analysis of high temperature hybrid CAES system eliminating the need for combustion in the traditional CAES system by incorporating two stages of heating through separate low temperature and high temperature TES units. Safaei *et al*.^14^ suggested that exporting the heat of compression to meet the space/water heating applications would contribute to the improvement of the overall efficiency. Simpore *et al*.^15^ performed dynamic simulation and observed the feasibility of utilizing CAES system integrated with a building in the case of PV power generation and the same was demonstrated. Trujillo *et al*.^16^ performed transient simulation analysis for CAES system without any thermal energy recovery unit and auxiliary fuel systems. Various studies^17–31^ were conducted to analyse the different configuations and performance of CAES system.

The first utility scale CAES system was commissioned at Huntorf, Germany in the year 1978, with the generation capacity of 290 MW, where the air is stored in the cavern with volume of 3, 10,000 m3 and with the operating pressure range of 46–72 bar. Subsequently, in the year 1991, another CAES based grid scale plant was commissioned at McIntosh, Alabama, USA with a generation capacity of 110 MW. In addition, the Energetix Group Ltd has considered this technology as a backup power supply (Compressed Air Battery – CAB) for standard and custom units from 3 kW to 3 MW which are made available for standby and uninterrupted power supply applications. This hybrid CAB facility is being adopted in British telecom sector as a third backup. This technology was also claimed by Telecom Italia (Italy), Eskom (South Africa) and Harris (US). However, the success rate of this technology remains difficult till today owing to the less overall turnaround efficiency of the system, arising as a result of complexities involved in the discharging process. It is a fact that without considerably augmenting the turnaround efficiency, attaining economic feasibility is questionable.

**Figure 1:**
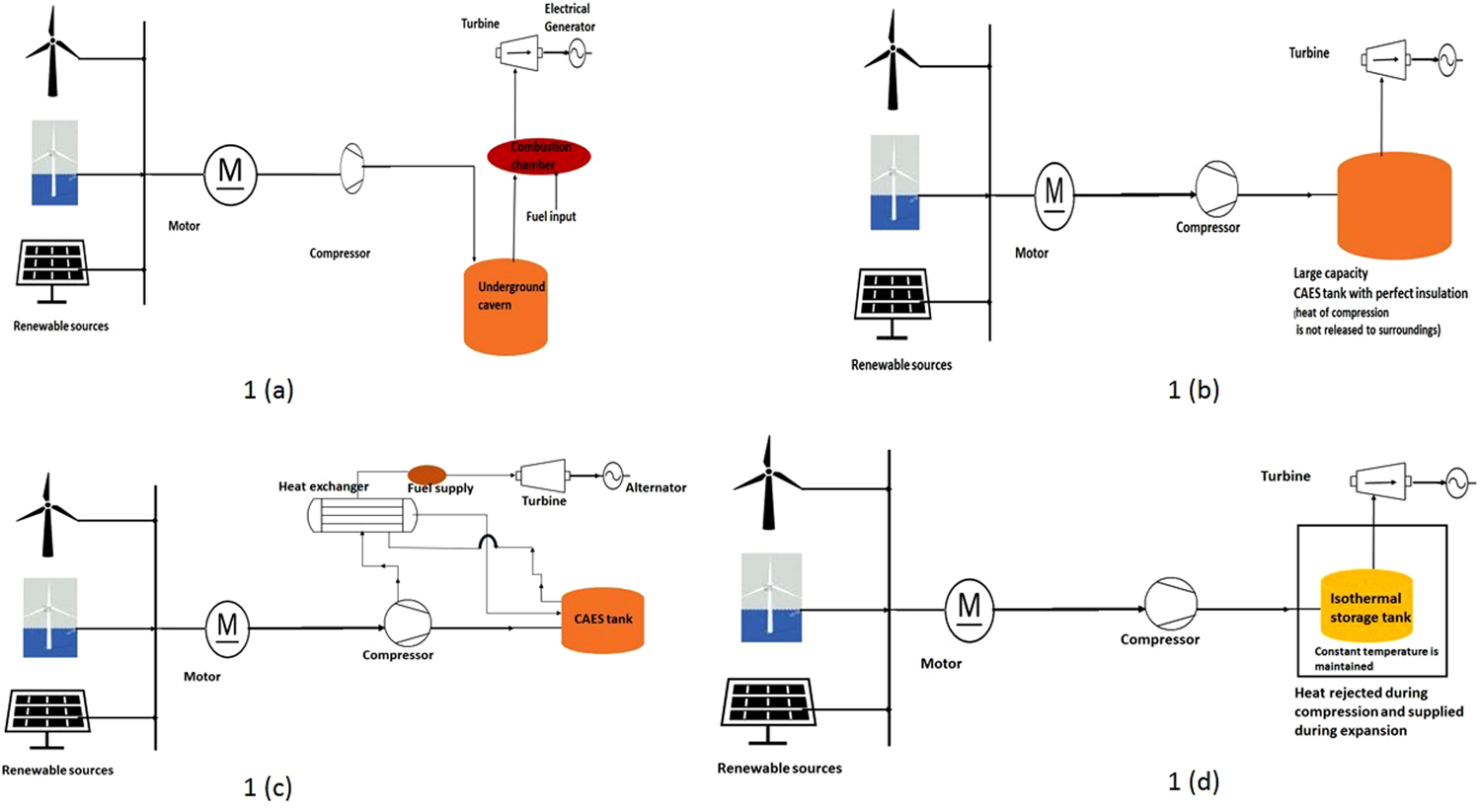
Various configurations of CAES system. (**a**) Diabatic storage system – Heat of compression is dissipated as waste. (**b**) Adiabatic Storage system – Heat of compression is not dissipated and stored as such in CAES storage tank. (**c**) Advanced adiabatic Storage system – Heat of compression is stored separately and utilized before expansion process. (**d**) Isothermal storage system – CAES tank is maintained at a constant temperature during charging and discharging processes.

In general, there are 4 major CAES configurations (i) Diabatic (ii) Adiabatic (iii) Advanced adiabatic and (iv) Isothermal system. (i) A Diabatic Compressed Air Energy Storage (D-CAES) system is an energy storage system based on the compression of air and storage in geological underground caverns. During the operation, the available electricity is used to compress air into a large storage system like salt cavern at depths of hundreds of meters and at higher pressure ranges depending on the depth of the cavern. The stored energy is released during the time of peak demand and the air is heated through combustion by means of natural gas or fuel and is expanded in a turbine to generate electricity. In diabatic CAES system, the heat of compression is not utilized and it is dissipated as waste. In the same system natural gas is used for combustion to heat the air before the expansion process during the discharging cycle. As discussed earlier, the existing two commercial CAES plants are being operated under this configuration which tends to generate a larger amount of GHG emissions. (ii) In the adiabatic system (A-CAES) the heat of compression is stored at a higher temperature and hence the generation of a larger quantity of power output can be expected. An adiabatic storage system does away with the intercooling during the compression process, and simply allows the air to heat up during compression, and stored in the CAES tank. However, in reality, more mass of air could be accumulated when the air temperature is minimum and hence in this configuration, in order to accommodate more mass of air at higher temperature the system demands for large volume of storage tank which ultimately escalate the investment cost up. (iii) In the advanced adiabatic storage system (AA-CAES) the heat of compression is stored in a thermal storage medium and, during the expansion process, this heat is retrieved for heating the compressed air and the additional heat is supplied by external sources to achieve higher power input. The initial investment required in this case will be little higher as a result of the additional thermal storage system and system heating involved. (iv) In isothermal storage configuration, (I-CAES) the temperature inside the storage tank is maintained constant by removing the heat during charging and supplying the heat during discharging process. All the above said process are represented schematically in Fig. 1.

Recently an attempt was made to summarize all the existing researches carried out by various scientists on Compressed Air Energy Storage system towards the efficient utilization of the intermittent Renewable Energy source. The consolidated report made through this effort was presented in (http://www.sciencedirect.com/science/article/pii/S1364032116301125). Several experimental attempts were also made in our lab during the last few years to understand the most suitable configuration to store energy in the compressed air form. Several research groups, across the globe have shifted their attention towards this potential area. Some of the below mentioned research findings have triggered our interest in a diversified manner that resulted in the proposal of this new concept of utilizing the naturally available thermal energy in the ocean/large water bodies for energy conversion, while utilizing the RE source integrated with CAES system.

- The research group headed by Yulong Ding, University of Birmingham, has started utilizing the cryogenic energy available during the expansion of compressed air for liquefaction of air.
- A Canadian start-up hydrostor utilizes a proprietary isobaric system built underground cavern for air storage, wherein water from ocean/sea is circulated to a CAES tank to maintain constant air pressure. Further, they have deployed the design made by Seamus Garvey, University of Nottingham, to put air bags under the sea at the required depth based on the pressure to be maintained (400–700 m) to utilize the static pressure of sea in a passive way.
- SustainX achieved isothermal cycling by utilizing the heat of compression available during the charging, which is stored in water and reutilizing the stored heat in the water during the expansion process in order to achieve isothermal compression and expansion. A similar kind of concept was utilized by LightSail by spraying water during compression and reutilizing that warm water spraying during the expansion process.
- The research team headed by Jihong Wang, University of Warwick, developed a multimode control strategy for air motor supply pressure control. Further, the patented technology mentioned in the link (https://www.google.ch/patents/US2863288) has claimed the invention of prothe control of air turbine governor for obtaining a constant air pressures supply/constant pressure ratio/constant speed for driving the machine (air motor). Further, yet another research team from the University of Birmingham has suggested a method called multipoint optimization. It is an optimization approach which is performed for a range of operating conditions to ensure acceptable performance levels in the turbine at this operating range.

The present research work focuses towards achieving higher turnaround efficiency through a novel concept of keeping the CAES in a large water bodies and thereby extracting the heat from the surrounding water bodies during the energy conversion process. A detailed thermodynamic analysis performed for the above said configurations along with the data analysis is explained in the methods provided at the end of this paper and the results are presented in the following section.

## Results

**Figure 2:**
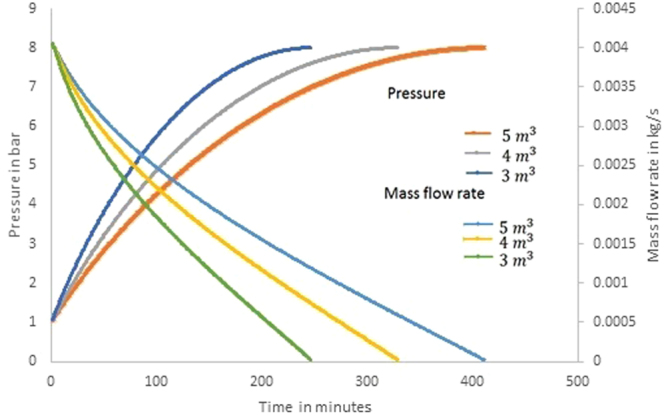
Variation of mass flow rate of air and pressure inside the storage tank during charging process.

Figure 2 shows the transient variation in the pressure and the mass flow rate of air in the CAES system for the analysis performed under different storage tank volumes (3 m^3^, 4 m^3^, and 5 m^3^). It should be noted that the work input available is 1 kW for the compression process and the process is isentropic. The increase in temperature of air at the end of compression is removed through a heat exchanger before it is allowed to enter the CAES tank and the tank is always maintained at isothermal condition. It is seen from the figure that the mass flow rate of air entering the tank decreases when the pressure inside the storage tank increases and as the work done by the compressor is prominently used to oppose the pressure resistance developed inside the tank.

Further, it is observed that the time required to develop 50% of the total pressure difference (i.e 1 bar − 4.5 bar) is 26.4% of the total time taken for the charging process in all the storage tank volumes considered. Similarly the time required for charging 75% of the total pressure difference (i.e 1 bar − 6.25 bar) is 47.1% of the total time taken in all the three cases. The remaining 25% of the pressure development (i.e. 6.25 bar – 8 bar) requires 53% of the total time required. This clearly explains that during the charging process the occurrence of first 50% pressure development takes place in an accelerated mode, the intermediate 50 to 75% pressure development takes place in a normal mode and the final 25% pressure development takes place in a decelerated mode. Hence, the exit pressure of the compressor should be at least 1.5 times (i.e. 12 bar in the present case) higher than the desired maximum pressure of air in the storage tank for an appreciable reduction in the work input by the compressor.

**Figure 3:**
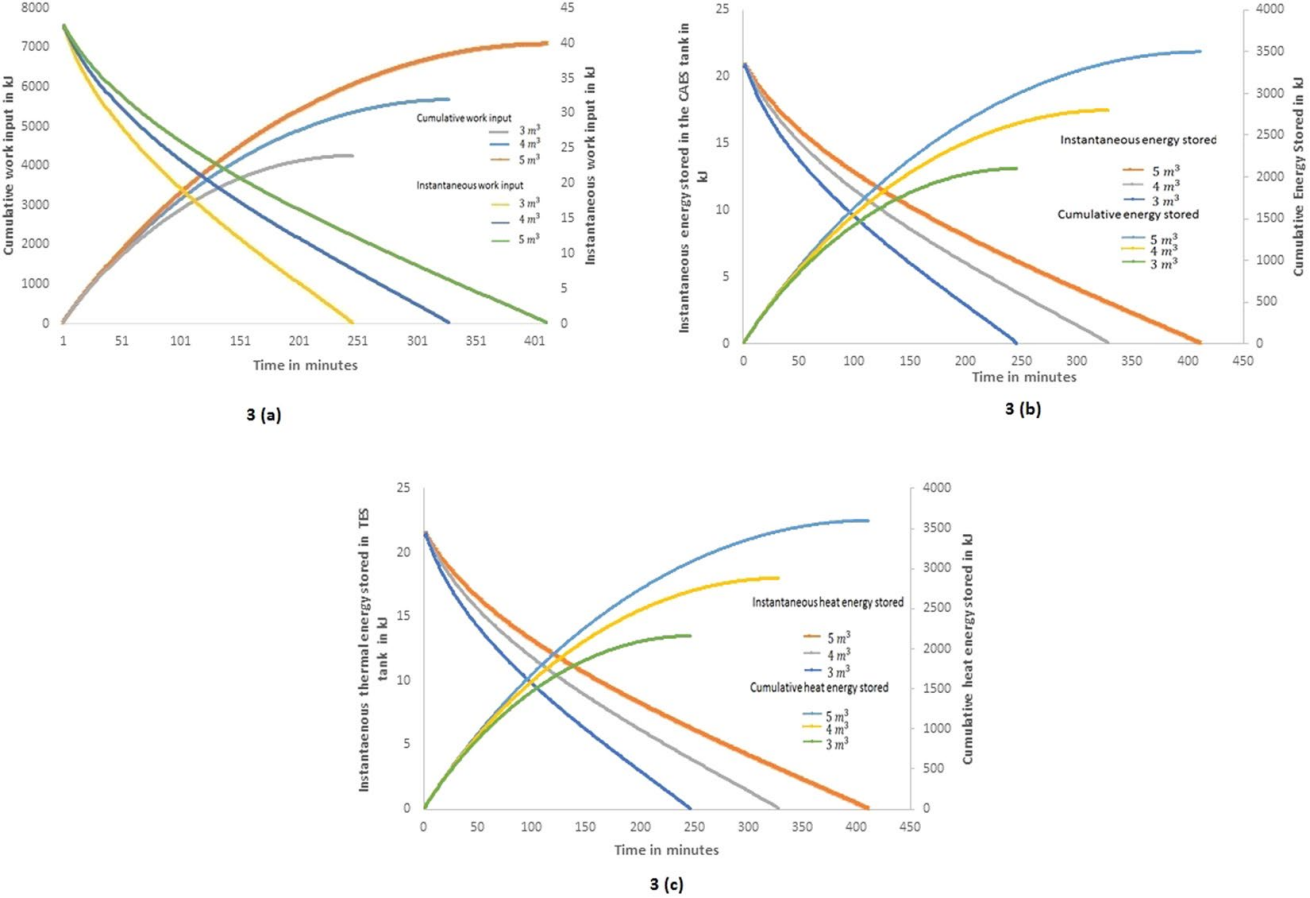
(**a**) Instantaneous and cumulative work input during charging process. (**b**) Instantaneous energy stored and cumulative energy stored in CAES tank during charging process. (**c**) Instantaneous and cumulative heat energy rejected from the compressed air and stored in the TES tank during charging process.

Figure 3 shows the various energy quantities associated during the charging process. During this process, the compression is considered as isentropic and the work input given to the compressor is converted into heat, resulting in increase in the temperature of air along with the increase in pressure. Figure 3(a) shows the instantaneous and cumulative work required to compress the air during the charging process. It is considered that all the components are operating with 100% efficiency and hence the energy/work quantities obtained at the end of charging process obeys the energy conservation law. The instantaneous work refers to heat transfer/work transfer evaluated in a small time interval and it is considered as 1 minute in the present transient analysis. It is seen from Fig. 3(a) that the instantaneous work input is 42 kJ/min during the start of charging process and it decreases to zero kJ/min within a duration of 249, 331 and 414 minutes respectively for the storage volumes of 3, 4 and 5 m^3^. The cumulative work required is 4260, 5680 and 7100 kJ respectively for the storage volumes of 3, 4 and 5 m^3^.

Since the compression process is considered isentropic (δQ = 0) the work input given to the compressor is utilized for increasing the internal energy of the air along with the increase in pressure. Figure 3(b) shows the instantaneous and cumulative pressure energy accumulated in the CAES tank at isothermal condition (atmospheric temperature). It is seen from the figure that the instantaneous rate of energy storage at the start of compression is 21 kJ/min, and it decreases to a minimum level within a duration of 249, 331and 414 minutes respectively for the storage volumes of 3, 4 and 5 m^3^. During these periods the cumulative pressure energy stored in the tank are 2100 kJ, 2800 kJ and 3397 kJ respectively for the storage volumes of 3, 4 and 5 m^3^. The difference in the cumulative energy in the various tanks are only due to the variation in the mass (volume) of air in the storage tank as the pressure in all the storage tank is same at 8 bar.

The heat energy available in the compressed air is removed and stored prior to allow this high pressure air to enter inside the CAES tank. Figure 3(c) shows the instantaneous thermal energy rejected from the compressed air which is stored in the thermal energy storage tank for maintaining isothermal condition in the CAES tank. It is seen from the figure that similar to the energy stored in the CAES tank, the instantaneous rate of energy storage at the start of compression in the TES tank is 21 kJ/min and it decreases to a minimum level within a duration of 249, 331 and 414 minutes respectively for the storage volumes of 3, 4 and 5 m^3^. Similarly the cumulative thermal energy stored in the TES tank is also 2160 kJ, 2880 kJ and 3702 kJ respectively for the storage volumes of 3, 4 and 5 m^3^. It is possible to utilize this heat energy either for heating application (or) to reheat the air before the expansion process. It is seen from the results that the cumulative sum of total compression pressure energy stored and the heat rejected which is stored in TES system are equal to the cumulative energy supplied as input at all instants.

**Figure 4:**
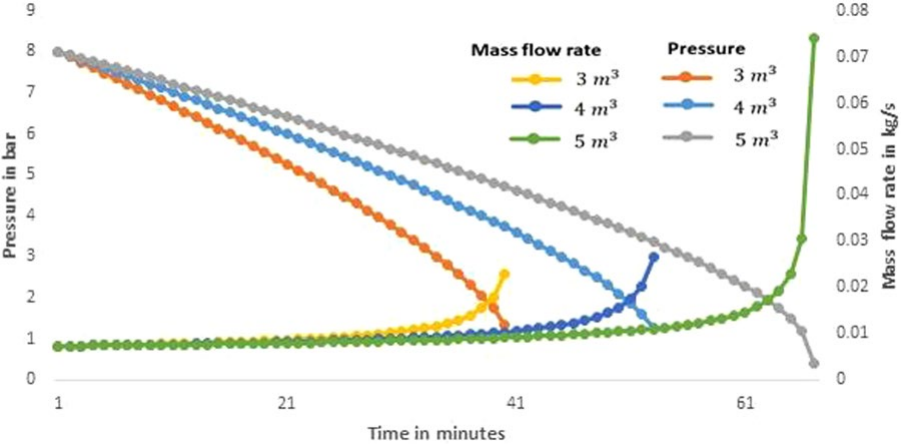
Variation in the pressure and mass flow rate during discharging process under different storage tank volumes.

Figure 4 shows the variations in the pressure and the mass flow rate required to achieve 1 kW constant power output from the turbine during the discharging process. It is observed from the figure that the expander operates for a duration of 40, 53 and 67 minutes respectively for the storage volumes of 3, 4 and 5 m^3^. Further it is seen from the figure that there is a slow increase in mass flow rate required till a certain point in all storage volumes and thereafter the mass flow rate required increases considerably which is not practically feasible due to the difficulty involved in the flow rate control arrangements. Hence, the extensive data were analysed and increase in mass flow rate required compared to the start of the expansion was found to be 28% when the pressure at the inlet of the turbine was 4.4 bar. The mass flow rate required was 34% higher when the inlet pressure was 4 bar. Nearly 30% variation in the mass flow rate control could be achieved easily through flow controls and also the expansion device can also be accommodated for the variations in the inlet pressure ratio of 1.8. Hence it is construed that the depth of discharging should not go below 4.4 bar which is 1.8 times lesser than the initial pressure in the storage tank.

**Figure 5:**
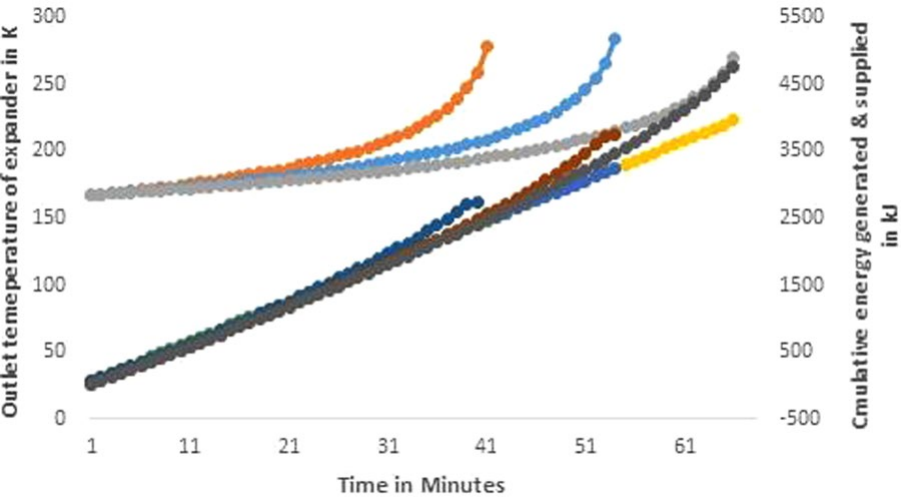
Variation in outlet temperature of the expander and the cumulative additional cool energy generated and the heat input supplied to maintain the isothermal condition in the storage tank for the constant generation of 1 kW power output under different storage tank volumes.

Figure 5 shows the variation in the outlet temperature from the expander during the discharging process and the cumulative cool energy available in the outlet air with respect to the ambient temperature conditions during the discharging process under constant load condition. Further, the results are presented to show the amount of heat energy supplied from the surrounding water to the storage TV of 3 m^3^, 4 m^3^, and 5 m^3^ to maintain the tank under isothermal condition during the expansion process. The quantities of total heat energy absorbed from the surrounding water at the end of discharging process are 2746 kJ, 3758 kJ and 4766 kJ for the TV of 3 m^3^, 4 m^3^, and 5 m^3^ respectively. It is possible to extract the required heat from the surrounding water by designing a large capacity heat exchanger. Thus the system has the ability of utilizing the free energy from the ocean and able to produce more work than the initial work potential in the storage tank.

The outlet temperature of air after the expansion process is initially at a very low level of 168 K. As the pressure in the tank decreases with respect to time, the outlet temperature also increases and, at the end of discharging process, the temperature will be close to 270 K. This cool energy is more precious than the power when some application is coupled with the CAES system for which a large quantity of cool energy is required. The quantities of cool energy that can be stored in a cool storage tank are 3960 kJ, 3240 kJ and 2460 kJ for the CAES TV of 3 m^3^, 4 m^3^, and 5 m^3^ respectively.

## Discussion

**Figure 6:**
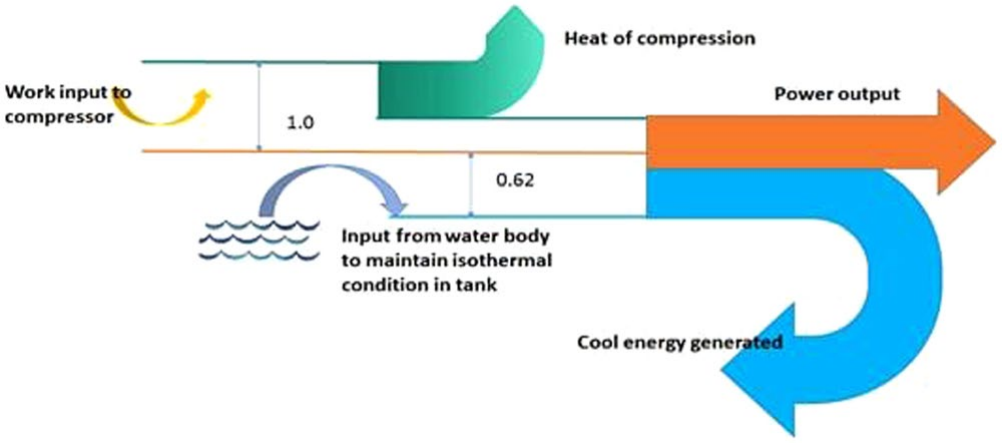
Sankey Diagram indicating the energy flow in the Isothermal CAES system.

The thermodynamic analysis performed in the present investigation assuming that all the components involved in the system were operated with 100% efficiency. Hence the energy balance could be shown using the first law of thermodynamics and the same is represented using the sankey diagram as shown in Fig. 6, considering one full charging and discharging cycle. It is seen from the sankey diagram that the output energy available in different forms such as heat energy, cool energy and work output cumulatively accounting 1.62 times the energy given as input to the compressor. This additional 62% energy is taken from the surrounding water at atmospheric conditions as detailed in Table 1. This additional net energy is possible when the heat transfer fluid works between the temperature limits above and below atmospheric temperature conditions, so that the energy available from the atmosphere can also be brought into the system. Though there is a possibility of energy multiplication with a factor of 1.62 theoretically, while implementing the system, in reality it involves several challenges owing to various practical constraints and efficiency factors involved in the system components.

**Table 1:** Energy balance showing harnessed energy from nature.

S: No	(Pressure range 1 bar–8 bar)	Charging Process				Discharging process				Net surplus output/Input
	Volume m^3^	Time of operation minutes	Work input	Heat rejected	Energy stored	Time of operation minutes	Cool energy generated	Power output	Heat input supplied	
1	3	249	4260	2160	2100	42	2400	2340	2433	0.62
2	4	331	5680	2880	2800	62	3180	3120	3731	0.62
3	5	414	7100	3702	3397	67	3960	3900	4766	0.62

**Figure 7:**
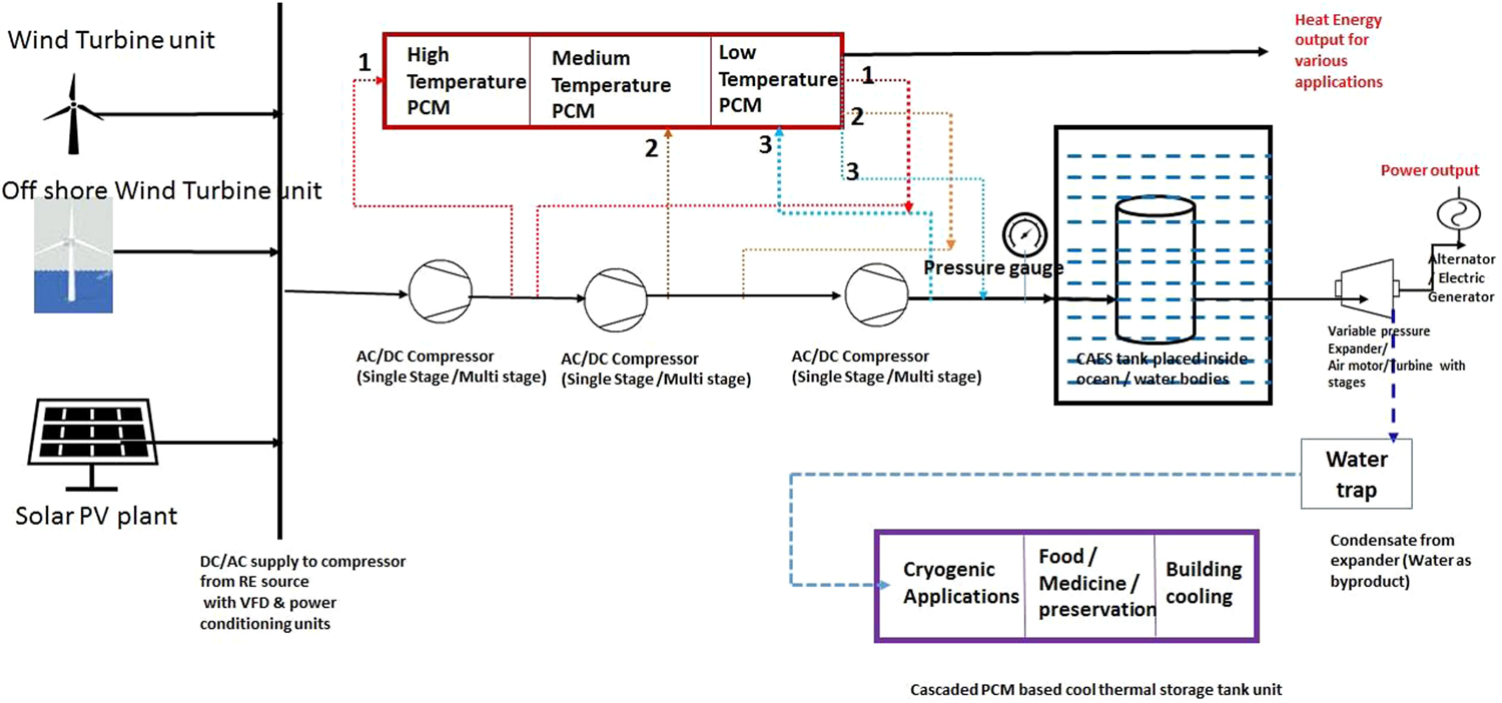
Illustration for the proposed isothermal CAES system with PCM based TES systems suitable for various applications.

The various challenges involved along with the possible solutions to maximize the overall turnaround efficiency are explained in this section. In the present system, for capturing the heat of compression generated during the charging process and the cool energy generated during the expansion process hot and cool thermal storage systems are required along with compressed air energy storage system. The charging and discharging cycle will not occur simultaneously in most instances of the system operation. Hence, the heat energy and cool energy generated during the process of operation cannot be utilized within the system by employing only the heat exchangers and thus the system demands the thermal storage systems. Once the storage system is employed there are several possible ways to utilize this cool and heat energy for other applications also when the usage for this energy has more value than the increased efficiency achievable within the system for cooling of air during compression process and heating of air before the expansion process. Figure 7, shows the applications possible with cascaded Phase Change Material (PCM) material based storage systems integrated with CAES system to achieve higher overall efficiency.

During the charging process since the objective is to store the compressed air at atmospheric temperature condition by keeping the CAES tank inside the water body/ocean in the case of large CAES system, the compressed air from outlet/intermediate stages from the compressor is allowed to pass through the heat exchanger integrated with PCM at various melting points based on the need for various applications before it is sent to the CAES tank. Thus the entire heat of compression can be retained in the storage system and the final compressed air which is entering into the CAES tank at a temperature closer to the atmospheric temperature condition will lose only very minimal heat energy during the period of storage. However, this hot storage tank should be insulated perfectly in order to retain the heat for longer duration. Further, during the above charging process by using proper controls and the selection of suitable multistage compressor incorporated with intercooling, the required work for the compression process could be reduced appreciably. It is possible to retain nearly 75% to 80% of the heat of compression at the required temperature so that the heat loss associated during the charging process would be less than 25%. Since the compressed air storage tank is kept inside the water body the losses associated during the storage process is negligible. The heat energy stored in the TES tank could be utilized for various other applications such as a thermal source for desalination units, industrial process heating and the low temperature heat source for building space heating.

During the discharging process, since there is a large thermal mass in the surrounding water, the transfer of heat to the storage tank can be achieved very easily when there is a reduction in temperature due to decrease in pressure. However, this may require a further heat transfer study to design the internal configurations of the CAES tank with extended surfaces compensating the lower heat transfer coefficients in the air side of the storage system. Thus, by achieving efficient heat transfer simultaneously during the discharging process, the abundant heat from the water body can be brought into the system when the expansion leads to a negative temperature in the CAES tank with respect to atmospheric conditions. Further, during the expansion process, the cool energy generated after expansion causes the liquefaction of air which may create a problem in the expansion process. However, this problem could be alleviated by allowing the fresh air through the cool storage system and removing the water particles before the air is allowed to pass through the compressor which reduces the work required by the compressor and also minimizes the problems associated with condensation of water during the expansion process. The cool energy stored can also be utilized for various applications based on the storage temperature. It should be noted that most of the countries located near the equator spend considerable electrical energy generated to produce cool energy. In the emerging RE scenario, if the CAES system is coupled with central cooling system of building/large food preservation applications, then the overall turnaround efficiency may be highly economical to commercialize this technology in the near future.

The major take away for the readers from the above study are,

- The proposed system will help the efficient deployment of RE sources by incorporating CAES system along with cool and hot thermal storage system and thereby improving the overall turnaround efficiency by harnessing large thermal energy from the naturally available water bodies where the CAES tank is placed and bringing the concept of polygeneration particularly in islands and offshore regions.
- The present study leads to understand the requirement of the exit pressure at the outlet of the compressor should be 1.5 times higher than the desired maximum pressure of air in the storage tank in order to reduce the compressor power required appreciably.
- The Depth of Discharging (DOD), pressure in the CAES tank should not be go below 1.8 times lesser than the initial pressure in the storage tank during the expansion process in order to reduce the mass flow rate variation within 30% which is achievable by the existing control strategies.

## Methods

### Configuration considered for analysis

**Figure 8:**
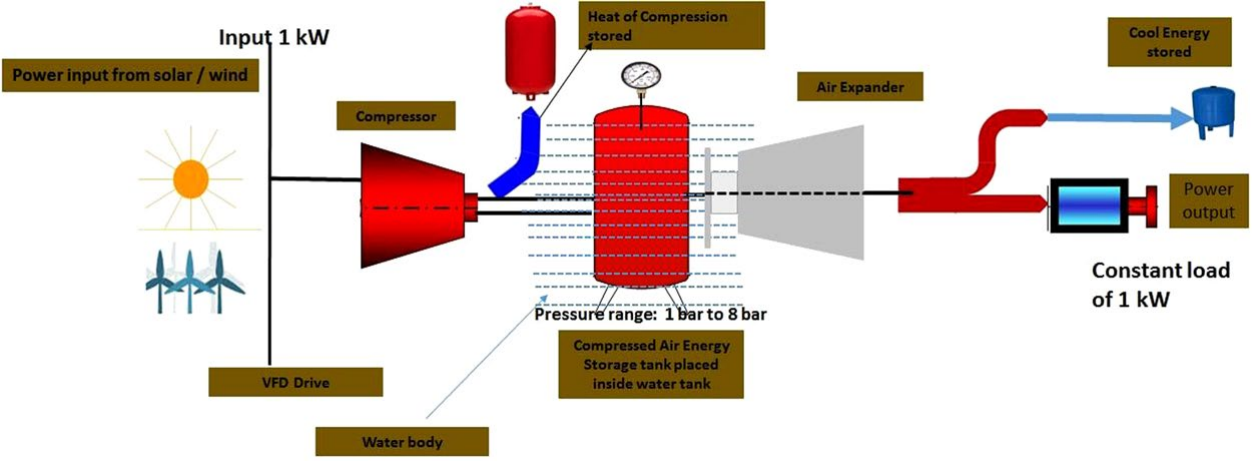
Isothermal CAES Configuration with improved turnaround efficiency (Configuration considered for analysis).

After undergoing a detailed study about the various configurations investigated and the recent advances attempted commercially by various companies, a novel passive approach is proposed to achieve a near isothermal condition by submerging/placing the compressed air storage tank under water bodies/ocean while the compressed air is discharged to generate power through air motor/air expander, thereby utilizing the infinite massive heat energy from the ocean in the energy conversion cycle. This concept is considered for a detailed thermodynamic analysis with certain operating conditions. Figure 8 shows the configuration considered for analysis in a microscale. It is assumed that the air from the atmosphere is compressed to 8 bar and the heat energy available in the compressed air is transferred to a hot thermal storage system before it is delivered to CAES tank. During the discharging process, while generating power using the expander certain quantity of additional cool energy is generated which is transferred to cool thermal energy storage tank. During this process the temperature of the air also gets reduced, when the pressure inside the tank decreases. However, as the tank is kept inside the large water body which acts as a thermal reservoir, it supplies the required heat instantaneously and maintains the temperature of the tank uniformly at 303 K. The methods to overcome the expected hurdles and the situations where this technology has potential applications in a medium and large scale are also discussed following the explanations elaborated in the results of the present analysis.

### Data Analysis

In order to analyse such isothermal storage systems, an in-depth thermodynamic approach is performed with transient data analysis. Normally any power transfer devices such as compressor and turbine are analysed under steady state conditions. However, the charging and discharging process involved in the CAES system makes it transient and hence the thermodynamic analysis is carried out with discrete time interval with a small time step. Further, the thermodynamic analysis is performed by assuming all the devices are operating with 100% efficiency and the heat transfer fluid as an ideal gas. The required work input to carry out the charging process is calculated by considering the initial power input of 1 kW in the present case study. Different thermodynamic variables and parameters are calculated for evaluating the instantaneous behaviour of the system, considering the ideal gas equation, energy and mass balance. Hence, the thermodynamic variables during charging process (temperature, specific volume, pressure and flow rate) are determined at each time step. Further, the parameters of interest like instantaneous energy stored in the compressed air storage system and the rejected heat of compression which is stored in the hot thermal storage tank are also quantified and presented. The above parameters are evaluated considering different storage tank volumes of 3 m^3^, 4 m^3^, and 5 m^3^. Similar to the charging phase, the first law is applied to the storage system in order to determine the relationship between its instantaneous pressure, instantaneous temperature and change in mass of the stored air under various storage tank volumes. In the discharging analysis it is assumed that the system delivers a constant power output of 1 kW at all time with the operating pressure range of 8 bar to 1 bar. The cool energy generated at every time instant and the energy harnessed from the water bodies to the storage tank are also calculated during the expansion. The thermodynamic equations (1–20) utilized to evaluate the various parameters involved during charging, storing and discharging process are presented in Table 2. The data analysis reveals a pathway towards utilizing the abundant free energy from the natural water bodies like the ocean for the efficient operation of CAES system and also unlocking the potential of renewable power generation.

**Table 2:** Thermodynamic formulae used in charging and discharging processes.

Parameter	Formula used	Eq. No
**Charging Process**		
***T***, ***P***, ***V***, ***m***, $\dot{{\boldsymbol{m}}}$ **and** ***ρ*** **represent the respective values evaluated at instantaneous time step i**		
Temperature of air leaving the compressor	$\frac{{T}_{oc}}{{T}_{ic}}={(\frac{{P}_{oc}}{{P}_{ic}})}^{\frac{{\rm{r}}-1}{{\rm{r}}}}\,$	1
Velocity at which the air is released from the compressor to the tank	$V=\sqrt{2{c}_{p}}\ast ({T}_{oc}-{T}_{t})$	2
Mass flow rate through the pipeline between the compressor and the tank	$\dot{m}=Compressor\,input\,power/({c}_{p}\ast ({T}_{oc}-{T}_{ic})$)	3
Density of the air present in the tank	${\rho }_{t}=({m}_{i-1}+\dot{m}\,\ast \,{\rm{\Delta }}\dot{t})/TV$	4
Mass in the tank	$m={\rho }_{t}\,\ast \,TV\,$	5
Temperature of the air in the tank at the next time step	${T}_{t}=\frac{[(\,{m}_{i-1}\ast {T}_{t,i-1})+(\dot{m}({\rm{\Delta }}t)\ast {T}_{oc})]}{{m}_{i-1}+\dot{m}({\rm{\Delta }}t)}$	6
Work input (W) during charging	${W}_{c}=[\dot{m}R({T}_{ic}-{T}_{oc})]/({\rm{\gamma }}-1)$	7
Pressure of the air in the tank	$P=\,{\rho }_{t}\ast R\ast {T}_{t}$	8
Cumulative work input	${W}_{Cum}=\,\sum _{i=1}^{N}\,{({W}_{c})}_{i}$	9
Instantaneous Energy Stored	${({E}_{inst})}_{isothermal}={({W}_{c})}_{i}-[({m}_{i}-{m}_{i-1})\ast {c}_{v}({T}_{t}-{T}_{amb})]$	10
Cumulative Energy stored	${E}_{cum}=\,\sum _{i=1}^{N}\,{({E}_{inst})}_{i}$	11
Instantaneous heat rejected (heat stored in hot TES tank)	${Q}_{hs-inst}={({W}_{c})}_{i}-({E}_{inst})$	12
Cumulative heat rejected (heat stored in hot TES tank)	${Q}_{hs-Cum}=\sum _{i=1}^{N}\,{({Q}_{hs-inst})}_{i}$	13
**Discharging Process**		
Instantaneous temperature at which the air leaves the expander	$\frac{{T}_{oe}}{{T}_{t}}={(\frac{{P}_{oe}}{{P}_{t}})}^{\frac{{\rm{r}}-1}{{\rm{r}}}}$	14
Instantaneous mass flow rate required for producing the power P	${\dot{m}}_{dis}=P/({c}_{p}\ast ({T}_{t}-{T}_{oe})$	15
Instantaneous mass available in the tank	$m={m}_{i-1}-({\dot{m}}_{dis}\ast {\rm{\Delta }}t)$	16
Instantaneous specific volume of the air in the tank	${{\vartheta }}_{t}=\frac{TV}{m}$	17
Pressure in the tank	${P}_{t}=({T}_{t{,}_{i-1}}\ast R)/{\vartheta }$	18
New temperature/Decrease in temperature	${T}_{t}=\frac{P}{{P}_{i-1}}\ast \,{T}_{i-1}$	19
Instantaneous Cool energy generated	${Q}_{cs-inst}=\,[{\dot{m}}_{dis}\ast {c}_{p}\ast ({T}_{amb}-\,{T}_{oe})\ast 60]$	20
Cumulative cool energy stored	${Q}_{cs-cum}=\,\sum _{i=1}^{N}{({Q}_{cs-inst})}_{i}$	21
Heat energy supplied to maintain isothermal temperature (free energy from ocean)	${Q}_{freeenergy}={m}_{t}\ast {c}_{v}\ast ({T}_{i-1}-{T}_{i})-TV\ast ({P}_{i-1}-{P}_{i})$	22
Polygeneration efficiency	${{\rm{\eta }}}_{Pol{y}_{aug.poly}}=({Q}_{cs-cum}+{Q}_{hs-Cum}+\,\sum _{i=1}^{N}\,{({W}_{c})}_{i})/(\,\sum _{i=1}^{N}{({W}_{c})}_{i})$	23
**Symbols:**		
T	—	Temperature in K
P	—	Pressure in bar
V	—	Velocity in m/s
$\dot{m}$	—	Mass flow rate in kg/s
*c*_*p*_	—	Specific heat of air at constant pressure in kJ/kg K
m	—	Mass of air in kg
W	—	Work input (or) Work output in kJ
R	—	Gas constant of air kJ/kg K
∆t	—	Time step size in seconds
*E*	—	Energy stored in kJ
*c*_*v*_	—	Specific heat of air at constant volume in kJ/kg K
Q	—	Heat quantity in kJ
*ϑ*	—	Specific volume in m^3^/kg
η	—	Efficiency in %
ɣ	—	Ratio of specific heats
**Subscripts & Superscripts:**		
oc	—	Outlet of compressor
ic	—	Inlet of compressor
t	—	Tank
i-1	—	previous time step
i	—	Current time step
inst	—	Instantaneous Energy in kJ
cum	—	Cumulative Energy in kJ
rej	—	Rejected
oe	—	outlet of expander
dis	—	discharging
N	—	Maximum number of time steps in a simulation trial
c	—	Compressor
e	—	Expander
amb	—	ambient condition
hs-inst	—	heat energy stored - instantaneously during charging
cs-inst	—	cool energy stored - instantaneously during discharging
hs-cum	—	heat energy stored - cumulative during charging
cs-cum	—	cool energy stored - cumulative during discharging
free energy	—	free energy from water bodies to maintain isothermal condition

### Data availability

The data that support the plots within this paper and other findings of this study are publicly available with the corresponding author and the same may be shared based upon the request.
